# Seroprevalence of Brucellosis and Associated Risk Factors among Indigenous Breeds of Goats in Wukari, Taraba State, Nigeria

**DOI:** 10.1155/2018/5257926

**Published:** 2018-11-01

**Authors:** Olaolu T. Olufemi, Danjuma B. Dantala, Paul A. Shinggu, Umesi A. Dike, Gbeminiyi R. Otolorin, Jivini A. Nwuku, Eyiojo B. T. Baba-Onoja, Tenmuso D. Jatau, Francis I. Amama

**Affiliations:** ^1^Department of Veterinary Public Health and Preventive Medicine, Faculty of Veterinary Medicine, University of Jos, Plateau State, Nigeria; ^2^Department of Animal Production and Health, Faculty of Agriculture and Life Sciences, Federal University Wukari, Taraba State, Nigeria

## Abstract

A cross-sectional study was conducted in Wukari, Taraba state, Nigeria, to determine the prevalence of* Brucella *antibodies and the risk factors associated with brucellosis in indigenous breeds of goats. A total of 386 goats were sampled from three political wards: Puje, Avyi, and Hospital: harvested sera samples were subjected to Rose Bengal Plate Test (RBPT). GraphPad Prism version 7.03 for Windows (GraphPad Software, La Jolla California, USA) was used to analyse the association between seroprevalence of brucellosis and age, sex, breed, location, and management system by using Chi square and Fisher's exact test as appropriate. Brucellosis was detected in all three wards: Puje; 15%, Avyi; 6.6%, and Hospital; 7.6%. A prevalence rate of 2.8%, 8%, 18.7%, and 1% was recorded for <20-month, 22-35-month, 36-45-month, and ≥46-55-month age categories, respectively (P < 0.05). Only 9.5% was observed for male animals while 9.8% was observed for female animals with no statistical difference between the males and females. Breed-specific seroprevalence yielded 7.4%, 5.4% 12%, 12.8%, and 11.6%, for Cross, West Africa Dwarf, Red Sokoto, Kano Brown, and Sahel breeds of goat, respectively. There is an evidence of brucellosis (9.6%) in Wukari L.G.A, Taraba State, and age is a risk factor for the disease in the study area. There is a need to enlighten the public on the zoonotic potentials and economic impacts of brucellosis.

## 1. Introduction

Brucellosis is recognised as one of the neglected tropical zoonotic diseases with a serious worldwide public health importance caused by members of the genus* Brucella* [1–3]. There are a total of 11 species [4]* with Brucella melitensis*,* B. suis*, and* B. abortus* in goats, pigs, and cattle, respectively, been the most important cause of morbidity and mortality worldwide [5]. Brucellosis in sheep, desert wood rats, dogs, pinnipeds, cetaceans, common voles (*Microtus arvalis*), and baboons is caused by* B. ovis*,* B. neonate*,* B. canis*,* B. pinnipedialis*,* B. ceti*,* B. microti,* and* B. papionis,* respectively.* B. inopinata* has been isolated in humans solely [6].

Brucellosis is endemic in ruminants in Nigeria with devastating economic and public health impacts ranging from retarded calving and decreased calving rate, culling due to infertility, decreased milk yield, abortions, still births, birth of weakly animals, and loss of man hours (in humans) to cost of treatment [7]. Several reports have been documented on farm animal brucellosis across different countries in Nigeria [7–11]. A prevalence of 16.1% was reported in Plateau State [12] while 8.6% was reported in Lagos State [13]. In the north-central, north-eastern south-western regions of Nigeria, 25.8%, 14%, and 0.86% prevalence were recorded, respectively, in goats [9, 14, 15]. This study is the first investigation of brucellosis in Wukari where most of the dwellers keep goats as a livestock at home thus increasing their likelihood of exposure to* Brucella melitensis*, the most pathogenic* Brucella* species that is known to affect humans [3].

Goats are a major source of meat supply in Wukari with a huge proportion of households keeping these animals as a source of income. Extensive management system is the predominant husbandry practice along with seasonal confinement where animals are tethered or allowed to graze freely during daylight in rainy season and allowed to fend for themselves in dry seasons.

In Wukari, Taraba state, the prevalence of brucellosis in goats have not been investigated and the factors associated with the infection is unknown. However, there are reports of ruminant brucellosis in other parts of the state: Jalingo (20%), Zing (19.9%) and Ardo-Kola (15.5%) [16]. Several reports of brucellosis in small ruminants across Nigeria exist [9, 17–23]. The aim of this study was to determine the seroprevalence of brucellosis and associated risk factors among indigenous breeds of goats in Wukari, Taraba State, Nigeria.

## 2. Methodology

### 2.1. Study Area

The study was conducted in Wukari metropolis of Taraba state, an ancient town in Kwararafa kingdom. Wukari is located in the guinea savanna region with both low forest and grassland vegetation. It lies within the coordinates latitude 7°51′ North and 9°47′ East. Most people in Wukari derive their livelihood from agriculture.

### 2.2. Study Design

A cross-sectional approach involving goats was conducted in the political wards in Wukari: Puje, Avyi, and Hospital. Data was collected over a period of three months across the three political wards in Wukari. It involved random sample collection from households and selected slaughter slabs following consent. Serological test and questionnaire survey were used as a tool for the determination the prevalence of brucellosis and assess the risk factors associated with age, sex, specie, and breed.

### 2.3. Sample Size and Sampling Procedure

With an expected prevalence of 20% [16] desired absolute precision (d) of 5% and confidence level of 95%, the sample size was calculated to be 236 [24]. A contingency of 63.5% was added and the sample was adjusted to 386.

Purposive sampling of the wards was carried out by aseptically collecting of 5 ml of blood by jugular venipuncture of each animal using 10 ml syringe and 21G needle following proper restraint. Blood samples were labeled and transported on ice pack to the laboratory where they were slanted and allowed to stand at room temperature overnight. Clear sera were harvested into labeled cryovials and stored at -20°C in the biology laboratory, Federal University Wukari, until needed for further analysis.

### 2.4. Laboratory Analysis

The sera were screened for antibodies against natural Brucella infection using Rose Bengal Plate-agglutination test (RBPT) standard protocol [25]. The Rose Bengal test antigen used was sourced from IDvet Innovative Diagnostics, France. Serum stored at −20°C was centrifuged to room temperature (37°C) using Centurion bench top centrifuge. Only 30 *μ*l of the serum sample was placed on a clean glass slide and an equal volume of antigen. A sterile plastic applicator was then used to mix the serum and the antigen thoroughly before slowly rocking it for 4 min to observe for agglutination. The result was appreciated by examining the degree of agglutination. Any visible agglutination was considered as positive while an absence of it was recorded negative.

### 2.5. Statistical Analysis

Data obtained were summarized and entered into Microsoft Excel 10 spreadsheet (Microsoft Corporation, Redmond, WA, USA) with the result for each test recorded. Descriptive and analytical statistics were used to explain the data. We evaluated significant differences between seroprevalence of brucellosis and age, sex breed, location, and management system by using Chi square and Fisher's exact test as appropriate. GraphPad Prism version 7.03 for Windows (GraphPad Software, La Jolla California, USA) was used to perform analysis. In all analysis, confidence level was at 95% and values of P<0.05 was considered significant.

## 3. Results

### 3.1. Seroprevalence of* Brucella* Antibodies in Goats Sampled in Wukari L.G.A Based on Wards

Among the 386 sera screened for* Brucella* antibodies, 37 (9.6%) were positive for Rose Bengal Plate Test (RBPT). Only 120 (13.1%), 122 (31.6%), and 144 (37.3%) goats were sampled in Puje, Avyi, and Hospital wards yielding a location specific seroprevalence of 18 (15%), 8 (6.6), and 11 (7.6%), respectively. There was no statistically significant association (p>0.05) between* Brucella* antibodies and the wards sampled (Table 1).

### 3.2. Seroprevalence of Antibodies Reactive to* Brucella* Antigen in Goats Sampled in Wukari Based on Age, Sex, and Breed

A total of 71 (18.4%), 199 (51.6%), 96 (24.9%), and 20 (5.1%) goats were sampled for the age categories, which yielded 2 (2.8%), 16 (8.0%), 18 (8.7%), and 1 (5.0%) positives for the ages of <20 months, 22-35 months, 36-45 months, and 46-55 months and above, respectively. There was a statistically significant difference (p<0.05) between age and the seroprevalence of brucellosis in goats sampled (Table 2) in Wukari L.G.A.

The sex specific seroprevalence revealed 9.5% and 9.8% for male (264) and female (122) goats, respectively (Table 2). There was no statistically significant association (P>0.05) between sex of goats sampled and the presence of brucellosis in Wukari L.G.A.

Breed-specific seroprevalence yielded 12.0%, 12.8%, 11.6%, 5.4%, and 7.4% for Red Sokoto, Kano brown, Sahel, West African Dwarf, and Cross breeds, from 100, 78, 43, 111, and 54 goats sampled, respectively. There was no statistically significant association (P>0.05) between breed of goats and the presence of brucellosis in Wukari L.G.A.

## 4. Discussion

The results of this study have shown that antibodies to brucellosis are present in goats in Wukari metropolis with an overall seroprevalence of 9.6%.

The seropositivity of 9.6% using Rose Bengal Plate Test (RBPT) is higher than that obtained by Ogugua* et al*., 2014, whose findings revealed an overall seroprevalence of 2.83% of brucellosis among goats screened across four states in Nigeria. This shows that brucellosis is endemic in Nigerian goats constituting a source of spread of the bacteria in spite of the paucity of data on brucellosis in goats in Wukari. The prevalence is higher than 1.9% in pastoral goats in eastern Ethiopia [26] and 2% reported in Uganda [27]. A higher overall prevalence of 18.2% in three selected local government areas in Taraba state is probably due to the inclusion of cattle and sheep although a high prevalence in goats (20%) can be attributed to the smaller sample size used in the study compared to this.

The seroprevalence of brucellosis in this study is lower than that reported by Al majali (27.7%) and Hamidullah et al., 2009 (34.88%), in Jordan, where positive sera with Rose Bengal Plate Test were further tested with complement fixation test for confirmation using standard* Brucella abortus* antigen. It is moreover lower than (16.10%) in northern Nigeria [28] and 45.75% reported in the outbreak of brucellosis in Abeokuta [20] and also (13.60%) in the herds of goats in north-eastern Ethiopia [29]. The seroprevalence obtained in this study is comparable with the findings of other researchers in Nigeria: 10.35% by Alhaji and Wungak (2013), 5.82% by Cadmus* et *al. (2006), 6% by Cadmus* et *al. (2010), and 6.28% by Ishola and Ogundipe [30]. The 9.6% seroprevalence in Wukari is probably due to the hardy nature of the* Brucella *organisms being able to survive harsh environmental condition remaining viable and infective [31–33]. Goats in the study area are often introduced into herds without prior quarantining. Furthermore, seropositivity within goats sampled in Puje ward was (15.0%) higher than those sampled in Hospital (7.6%) and Avyi (6.6%). However it can be observed that the seroprevalence recorded in Puje is due to urbanization and densely populated pattern of settlement which is relatively higher than in Hospital and Avyi. This could be due to different source of purchase [12].

This study recorded a statistically significant association (p<0.05) between age specific prevalence and* Brucella *antibodies, with goats between 36 and 45 months old having the highest prevalence (18.7%). This could probably be because susceptibility to brucellosis increases after sexual maturity and pregnancy. Exposure also occurs during mating and feeding on contaminated pasture and water. This finding is similar to the reports of Bertu et al. (2010), Farouk* et al*. [34], Bala (2013), and Zubairu* et al*., 2014. The higher seroprevalence of* Brucella *antibodies in males observed in this study is probably because of the innate aggression and high libido in males. Traditionally, a buck is exposed to more females during mating increasing its chances of infection. This finding disagrees with the reports of Adugba et al. (2006) and Ashenafi et al. [35], but agrees with other studies [10, 14].

The breed-specific seroprevalence revealed no significant association (p<0.05) between seropositivity and breed of the goats sampled. Although* Brucella* infection is not breed specific [36, 37], the highest seroprevalence was recorded among the Red Sokoto breed when compared to Kano brown, Sahel, West African Dwarf, and Cross breeds. This report is in line with Junaidu et al. (2010) and Tijjani et al. (2009) who reported highest prevalence of brucellosis in Red Sokoto breed. This finding however is in contrast with the findings of Dogo et al., 2016.

Goats purchased from the markets recorded higher prevalence compared to those inherited. Goats are obtained from different areas and locations [12]. This report is similar to reports in Adamawa, Kaduna, and Kano States, respectively [7]. There is a high possibility that infections will be maintained longer in inherited animals. On the abortion history, 11.8% and 10.4% indicate the rates of abortion. However, abortion is strongly associated with brucellosis; similar findings had been reported [18, 38, 39]. Seroprevalence of brucellosis and antibodies according to respondents to retained placenta showed that a total 43 and 133 recorded for Yes and No. The specific rates of 11.6% and 10.5% from the respondents show that retained placenta is associated with brucellosis strongly.

This study investigated the management systems of animals, intensive, semi-intensive, and extensive system. The highest rate recorded in semi-intensive system is probably due to out-grazing with different herds and shedding of the disease in pen upon return from pasture grazing and mating.

The highest seroprevalence observed when animals are fed collectively could be as a result of contamination of feed, feeding trough, and other fomites. This study recorded 33 and 143 for those who consume goats' milk and those who do not; specific rate shows that 9.1% consumed goats' milk while 11.2% do not. However consumers of goat milk are at high risk of brucellosis.

The rate at which aborted materials were disposed is high by burning followed by buried and thrown into bush at a specific rate of 25.0%, 11.8%, 9.2%, and 4.3%. This is due to picking by carnivorous animals (dog). Hand washing practiced showed 124 and 52 respondents who washed their hands and those who do not; specific rate indicates that 12.9% and 7.7% were among those who practiced hand washing and those who do care to wash their hands. This is probably due to odour of the animals and their discharge.

## 5. Conclusion

There is an evidence of brucellosis in Wukari with an overall seroprevalence of 9.6%. This constitutes high potential risk of infection and serious public health significance. It poses great risks to the livestock population, livestock owners, abattoir workers, meat vendors, livestock marketers, and professional animal health workers in Wukari L.G.A, Taraba State. Age is a risk factor to* Brucella* infection in goats in the study area. There is need for awareness on the economic and public health implications of brucellosis.

## Figures and Tables

**Table 1:** Seroprevalence of antibodies to brucellosis in goats sampled in Wukari based on wards.

**Wards**	**Number of sera tested**	**Number of positive (%)**	**Odds ratio**	**95% CI**	**P-value**
			**(OR)**	**On OR**	
**Puje **	120	18 (15)	1.00		0.050
**Avyi**	122	8 (6.6)	2.51	1.09-5.74	-
**Hospital**	144	11 (7.6)	1.65	0.74-3.59	-

Total	386	37 (9.6)			

**Table 2:** Seroprevalence of antibodies to *Brucella* in goats sample in Wukari based on age, sex, and breeds.

**VARIABLES**	**NUMBER OF SERA TESTED**	**NUMBER OF POSITIVE (%)**	**ODDS RATIO**	**95% CI**	**P-VALUE**
			**(OR)**	**On OR**	
**AGE**					
<20 months	71	2 (2.8)	1.71	0.11-15.2	0.007
22-35 months	199	16 (8.0)	0.60	0.06-3.51	
36-45 months	96	18 (18.7)	0.23	0.02-1.33	
46-55 months and above	20	1 (5.0)	1.00		
**SEX**					
Male	264	25 (9.5)	0.96	0.48-1.94	0.909
Female	122	12 (9.8)			
**BREED**					
Red Sokoto	100	12 (12.0)	1.00		0.360
Kano Brown	78	10 (12.8)	0.93	0.40-2.31	
Sahel	43	5 (11.6)	1.04	0.33-2.81	
West African Dwarf goat	111	6 (5.4)	2.39	0.87-6.53	
Cross	54	4 (7.4)	1.70	0.58-5.02	

**TOTAL**	386	37 (9.6)			

## Data Availability

All data used to support the findings are included within the article.
